# Interference of Particles with Fermionic Internal Degrees of Freedom

**DOI:** 10.3390/e26060449

**Published:** 2024-05-26

**Authors:** Jerzy Dajka

**Affiliations:** 1 Institute of Physics, University of Silesia in Katowice, 40-007 Katowice, Poland; jerzy.dajka@us.edu.pl; 2 The Professor Tadeusz Widła Interdisciplinary Research Centre for Forensic Science and Legislation, University of Silesia in Katowice, 40-007 Katowice, Poland

**Keywords:** interference, fermions, Hubbard dimer, quantum control

## Abstract

The interference of fermionic particles, specifically molecules comprising a small number of fermions, in a Mach–Zehnder interferometer is being investigated under the influence of both classical and non-classical external controls. The aim is to identify control strategies that can elucidate the relationship between the interference pattern and the characteristics of internal fermion–fermion interactions.

## 1. Introduction

Interference experiments serve as a powerful tool to unveil and observe the most quantum, often counter-intuitive, features of nature. The Mach–Zehnder type of interferometry stands as a fundamental example wherein a beam of particles splits, traverses two paths, and subsequently reunites to exhibit interference. In contemporary research, interference phenomena are not limited to simple particles like photons but extend to entities potentially endowed with internal degrees of freedom [1]. In such cases, the output intensity along a given path depends not only on the interferometer configuration but also on the intricate details of the time evolution of internal degrees of freedom.

Fermions, as fundamental constituents of matter, naturally feature in discussions regarding interfering objects. Fermionic systems, with their distinctive entanglement and transport properties, often serve as primary candidates for nano-solid systems [2,3].

The relationship between the microscopic description of multi-particle low-dimensional nanosystems and the transport properties of conducting electrons has been extensively explored. These investigations delve into considerations of inter-particle interactions and the complex connection between transport properties in nanorings [4,5,6,7,8,9].

Hubbard-type fermionic systems offer a foundational model in quantum many-body physics, where the interplay between quantum-mechanical hopping (tunneling) and particle–particle interactions plays a pivotal role [3]. Experimental realizations of Hubbard-type systems using ultra-cold fermionic atoms in optical lattices have been achieved, demonstrating high controllability in such systems [9,10,11,12]. Due to its conceptual simplicity, fermionic dimer model is useful in investigating theoretical methods [13]. Recently, the two-site Hubbard model was realized experimentally using ultracold techniques [14].

In this paper, we investigate Mach–Zehnder interferometry involving the smallest fermionic molecules, specifically Hubbard-type dimers, influenced by a controlling field present in one arm of the interferometer. We explore two distinct open-loop control strategies: the first being electrostatic, purely classical, achievable, for example, through a classical gate potential in one of the interferometer arms. The second strategy involves a fundamentally quantum-mechanical approach, where non-classical electromagnetic fields act on fermionic molecules within one of the interferometer arms. The effect of two-path interference can be quantified in terms of output intensities along the paths. Our objective is to establish a relationship between the output intensity and selected characteristics of both the interfering fermionic molecules and the tailored control parameters. Specifically, we investigate whether the fermion–fermion interaction, including both on-site and inter-site (i.e., non-local) interactions, is correlated with the interference pattern. Additionally, we identify features of non-classical open-loop controls that lead to desired output intensity values, such as the initial preparation (state) of electromagnetic radiation or the strength of coupling between the controlling field and fermionic dimers. Our paper is structured as follows: In Section 2 (Materials and Methods), we formulate a model of fermionic Mach–Zehnder interference in the presence of two types of controls: the first being an electrostatic gate potential affecting solely fermion–fermion interactions, and the second being a non-classical single-mode electromagnetic field coupled to the spin of fermionic molecules. Subsequently, in the Results section, we present our observations resulting from the chosen dynamical model. Finally, in the last section, we discuss the implications of our findings.

## 2. Materials and Methods

In our study, we concentrate on the interference of one of the simplest fermionic systems, namely dimers. The dimer is characterized by an “extended” two-site Hubbard Hamiltonian that accounts for the inter-site Coulomb interaction:

(1) $$\begin{array}{cc} H(V_{C},J_{C1},J_{C2}) & =-\tau \sum_{s\in \{↑,↓\}}\left(c_{1s}^{†}c_{2s}+h.c.\right)+V_{C}\sum_{j=1}^{2}n_{j↑}n_{j↓}+\\ & +J_{C1}\left(n_{1↑}n_{2↑}+n_{1↓}n_{2↓}\right)+J_{C2}\left(n_{1↑}n_{2↓}+n_{1↓}n_{2↑}\right) \end{array}$$

written in terms of fermionic creation $c_{js}^{†}$ and annihilation $c_{js}$ operators where $n_{js}=c_{js}^{†}c_{js}$ for j=1,2 and s=↑,↓. As the hopping integral τ Equation (1) establishes the characteristic energy (or time) scale, further we set τ=1.

In Equation (1), the first term represents the electronic hopping set a unit of energy throughout the paper serving as a natural unit of time. The term proportional to $V_{C}$ describes the on-site Coulomb interaction. Additionally, the term involving $J_{C1,2}$ extends the Hubbard model [2] by incorporating a spin-dependent inter-site Coulomb interaction [15,16]. As the Hubbard model [2] is specifically designed to describe systems that exhibit both electronic (transport) and magnetic (spin) properties, it is pertinent to mention

(2) $$\begin{array}{ccc} S_{z}^{\left(j\right)} & = & \frac{1}{2}\left(n_{j↑}-n_{j↓}\right),\;\;j=1,2 \end{array}$$

describing the *z*-component of spin operator of each of the dimer sites.

We consider interference of fermionic dimers in a conventional Mach–Zehnder interferometer [17] (further abbreviated as MZ interferometer) of two paths spanning two-dimensional Hilbert space $\tilde{H}=\{|\tilde{0}\rangle ,|\tilde{1}\rangle \}$ where each of the basis vectors indicates particle moving along a certain path. On this basis, beam splitters *B*, mirrors *M*, and relative phase χ shift *F* are represented by

(3) $$M=\left(\begin{array}{cc} 0 & 1\\ 1 & 0 \end{array}\right),B=\frac{1}{\sqrt{2}}\left(\begin{array}{cc} 1 & 1\\ 1 & 1 \end{array}\right),F=\left(\begin{array}{cc} e^{i\chi } & 0\\ 0 & 1 \end{array}\right).$$

As the particle passing through the interferometer is a fermionic dimer, the total space of the system is a tensor product $\tilde{H}⊗H_{F}⊗H_{E}$, where $H_{F}$ and $H_{E}$ denote state spaces of fermionic system and quantum controlling quantum bosonic field used in further considerations. An action of the interferometer is then given by a unitary operation:

(4) $$\begin{array}{ccc} U_{MZ} & = & \tilde{B}\tilde{M}U\tilde{B} \end{array}$$

where $\tilde{X}=X⊗I_{F}⊗I_{E}$ for X=M,B and $I_{F,E}$ is an identity operator acting on fermionic and controlling bosonic environment space, respectively. The role played by internal degrees of freedom is encoded in U which reads as follows:

(5) $$U=\left(\begin{array}{cc} 0 & 0\\ 0 & 1 \end{array}\right)⊗\exp\left(-iHt\right)+\left(\begin{array}{cc} e^{i\chi } & 0\\ 0 & 0 \end{array}\right)⊗\exp\left(-iH^{0}t\right)$$

Equation (5) possesses a clear interpretation: the time evolution of a particle (fermionic dimer) passing through the interferometer varies between different arms of the device because it is generated by different Hamiltonians $H,H^{0}$. We assume that the incident fermionic dimer entering the interferometer travels along $|\tilde{0}\rangle$. Fermionic degrees of freedom in both arms of the interferometer undergo different evolution given by |ψ(t)〉=exp(−iHt)ψ(0)〉 and $|\phi \left(t\right)\rangle =\exp\left(-iH^{0}t\right)\phi \left(0\right)\rangle$. The output intensity along $|\tilde{0}\rangle$ is, for the phase shift equal χ and the time required for passing trough the interferometer *t*, given by [17]:

(6) $$\begin{array}{ccc} P & = & \frac{1}{2}\left[1+|\Xi |\cos(\mathrm{Arg}(\Xi )+\chi )\right] \end{array}$$

where Ξ=〈ϕ(t)|ψ(t)〉 reads as

(7) $$\begin{array}{ccc} \Xi & = & \langle \exp\left(-iH^{0}t\right)\phi \left(0\right)|\exp\left(-iHt\right)\psi \left(0\right)\rangle \end{array}$$

and obviously, $\left|\psi \left(0\right)\rangle =|\phi \left(0\right)\rangle \equiv \right|\psi_{i}\rangle$ is an initial state of the dimer molecule. Time instant *t* in Equation (6) when interfere occurs is assumed to be larger than a typical time characterized evolution of the dimer molecule. For the assumed value τ=1 of hopping in Equation (1), we set t=30.

We examine two qualitatively distinct control strategies. The first is purely classical, induced in different arms of the MZ interferometer by distinct electrostatic gate potentials, resulting in varying values of on-site and/or inter-site Coulomb interactions in Equation (1). In one arm, the time evolution of the fermionic dimer is governed by the Hamiltonian:

(8) $$\begin{array}{ccc} H^{0} & = & H(V_{C}^{0},0,0) \end{array}$$

whereas in the second arm of the MZ interferometer the time evolution is generated by

(9) $$\begin{array}{ccc} H & = & H(V_{C},J_{C1},J_{C2}) \end{array}$$

The second control strategy is quantum in nature. We posit that the fermionic dimer in one of the two arms of the MZ interferometer interacts with a single-mode bosonic system representing, for example, non-classical radiation [18,19]. The total Hamiltonian in that arm is expressed as follows:

(10) $$H=H\left(V_{C},J_{C1},J_{C2}\right)+\omega a^{†}a+\kappa \left(S_{z}^{\left(1\right)}+\epsilon S_{z}^{\left(2\right)}\right)⊗\left(a^{†}+a\right)$$

where $a,a^{†}$ are bosonic (commuting) operators generating Heisenberg–Weyl algebra [19,20]. Bosonic field is coupled to the dimer via its spin operators Equation (2) where κ is a coupling strength. Let us emphasize that Equation (10) enables the consideration of potentially non-equal coupling to dimer sites for ϵ≠1 in the image of Friedrichs-type modelling of multi-level systems with only one of the levels affected by the environment. Here, ϵ, as an additional parameter, allows us to remove one of symmetries present in the system.

For both control strategies and fermionic vacuum state |Ω〉 (defined by the condition $c_{is}|\Omega \rangle =0$ for i=1,2 and s=↑,↓), we examine two initial preparations of fermionic dimers:

(11) $$\begin{array}{ccc} |\psi_{i}^{a}\rangle & = & c_{1↑}^{†}c_{2↓}^{†}|\Omega \rangle \end{array}$$

(12) $$\begin{array}{ccc} |\psi_{i}^{b}\rangle & = & c_{1↑}^{†}c_{2↑}^{†}|\Omega \rangle \end{array}$$

which are different in their total spin. For quantum control, Equation (10), the initial preparation requires specification of the bosonic initial state:

(13) $$\begin{array}{ccc} |\Psi_{i}^{a,b}\rangle & = & |\psi_{i}^{a,b}\rangle ⊗|N\rangle \end{array}$$

(14) $$\begin{array}{ccc} |N\rangle & = & \frac{{\left(a^{†}\right)}^{n}}{\sqrt{N!}}|0\rangle ,\;\;\;N=0,1,2,\ldots \end{array}$$

with the bosonic vacuum state |N=0〉 defined by the condition a|0〉=0. Furthermore, we restrict our focus to number eigenstates that are not only non-classical in a quantum optical sense [20] but also do not introduce additional phase to the system.

To manipulate the properties of nanosystems, both the electronic and magnetic characteristics of Hubbard-type systems offer avenues for implementing effective control strategies through the application of classical or non-classical electromagnetic fields. These control strategies can be static, such as electrostatic gating, or dynamic, involving non-classical approaches [9,21]. However, dealing with time-dependent quantum systems in an exact manner presents challenges. In this paper, all the numerical calculations required to compute the time evolution of the fermionic systems are performed using QuTiP Version 4.7.6. QuTiP is an open-source software package specifically designed for simulating the dynamics of open quantum systems [22,23]. In our calculations, we used the ’mesolve’ solver implemented in QuTip. Heisenberg–Weyl algebra of bosonic creation and annihilation operators and number eigenstates are directly implemented in QuTiP. Fermionic operators were constructed with the help of Pauli matrices also provided by QuTiP.

## 3. Results

**Figure 1:**
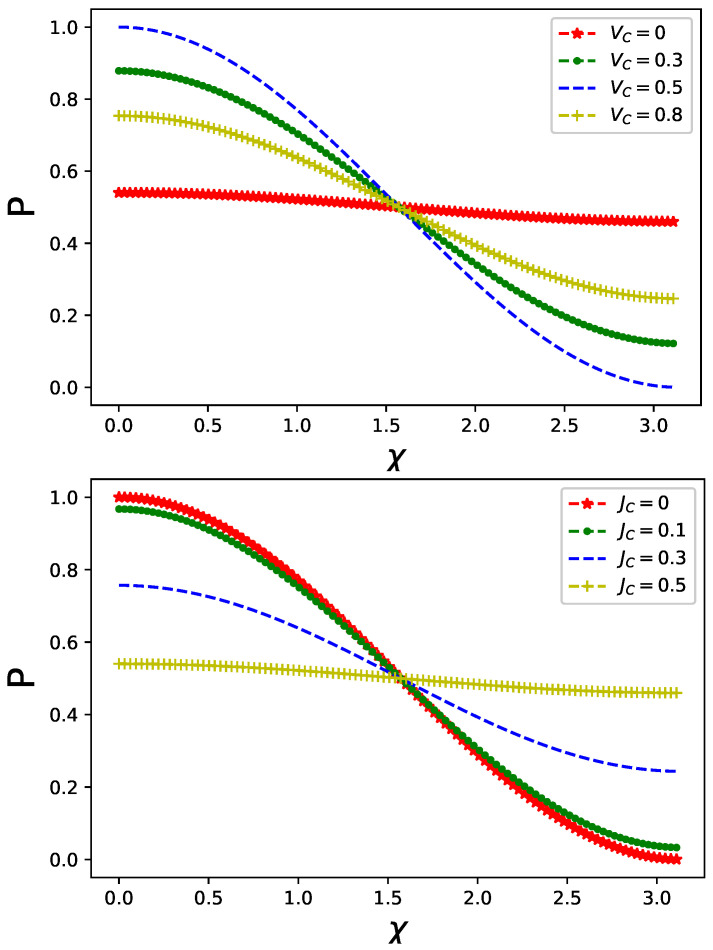

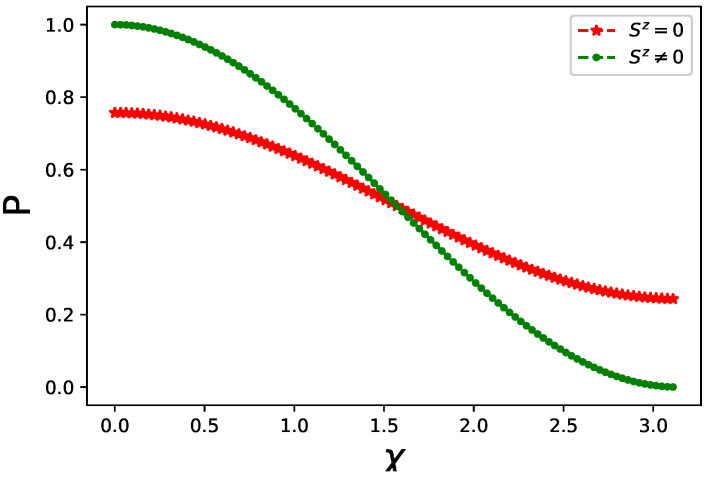
Output intensity Equation (6) for different values of on-site Coulomb interaction (**top panel**) and inter-site Coulomb interaction (**middle panel**) in one arm of the MZ interferometer. In the second arm, only an on-site interaction is present with $V_{C}^{0}=0.5$. Interfering fermionic systems are initially prepared in a spin-zero state $|\psi_{i}^{a}\rangle$ Equation (11). (**Bottom panel**): output intensity Equation (6) for two fermionic initial states $|\psi_{i}^{a,b}\rangle$ characterized by vanishing or non-vanishing value of the spin z-component with $V_{C}=0.2$ and $J_{C}=0$.

Interference pattern represented by the output intensity *P* in Equation (6) in the presence of classical static control, i.e., electrostatic gate potential present in one of the arms of the MZ interferometer, is depicted in the top and middle panels of Figure 1 for a difference in on-site and inter-site Coulomb interaction, respectively.

The first observation is that any difference in Coulomb interaction, both on-site (see top panel of Figure 1) and inter-site (see middle panel of Figure 1), affects the output intensity, resulting in its reduction compared to the case with no static control applied in one of the arms of the interferometer.

It is best visible for $V_{C}=V_{C}^{0}=0.5$ when an overlap between fermionic states in Equation (6) approaches its maximal value. The difference in time evolution generators in different arms of the interferometer result in a reduction in this overlap that corresponds to a decreasing value of *P* presented in the top and middle panels of Figure 1. This effect is most apparent for initial preparations of a fermionic component of an overall state characterized by vanishing z-component of spin as it can be inferred from the bottom panel of Figure 1.

**Figure 2:**
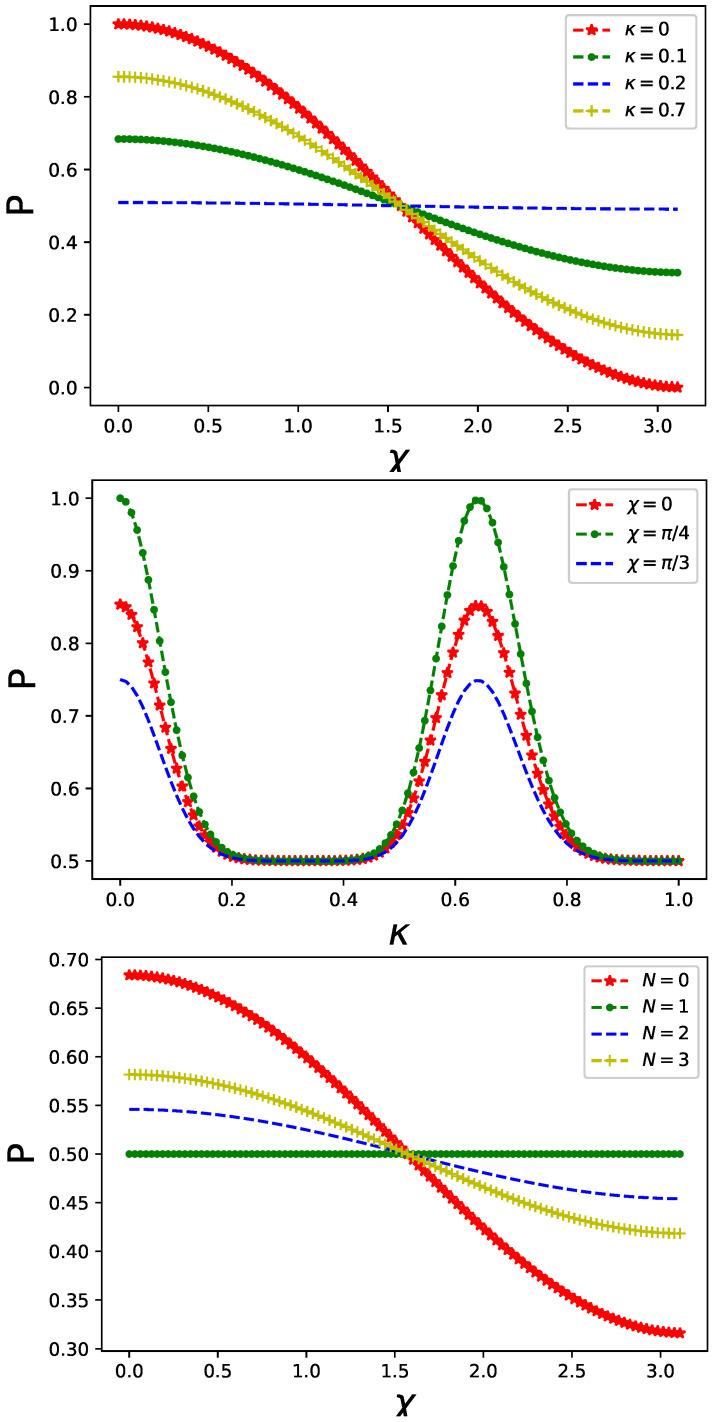
Output intensity Equation (6) in a presence of non-classical control affecting one arm of the MZ interferometer for different values of coupling strength κ Equation (10) (**top panel**) and for different values of χ in a phase gate (**middle panel**). Fermionic dimer and controlling boson are prepared in $|\psi_{i}^{b}\rangle ⊗|N=0\rangle$ state. Only an on-site Coulomb interaction is included with $V_{C}=0.5$ in both arms of interferometer. Coupling of the controlling boson and fermionic degree of freedom is symmetric with ϵ=1 in Equation (10). Output intensity Equation (6) for different initial states $|\psi_{i}^{b}\rangle ⊗|N\rangle$ is compared in the (**bottom panel**).

**Figure 3:**
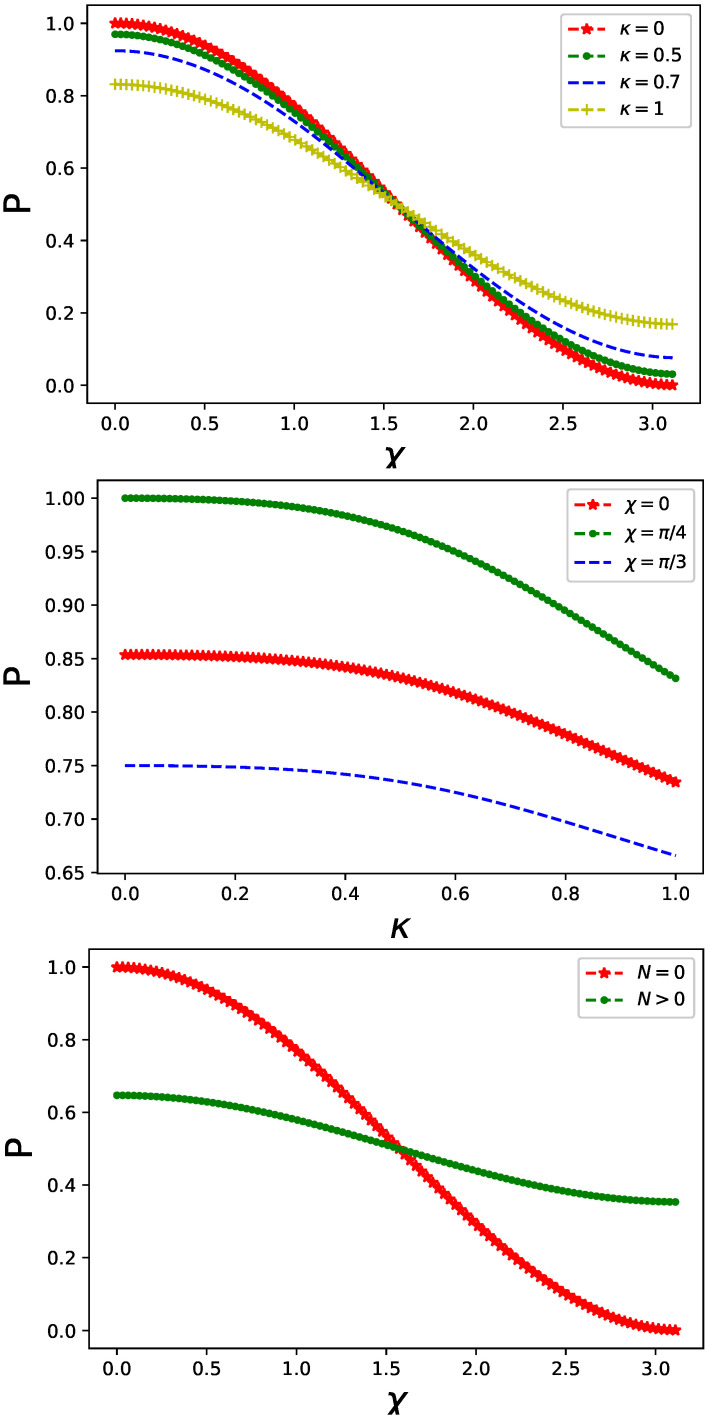
Output intensity Equation (6) in a presence of non-classical control affecting one arm of the MZ interferometer for different values of coupling strength κ Equation (10) (**top panel**) and for different values of χ in a phase gate (**middle panel**). Fermionic dimer and controlling boson are prepared in $|\psi_{i}^{a}\rangle ⊗|N=0\rangle$ state. Only the on-site Coulomb interaction is included with $V_{C}=0.5$ in both arms of the interferometer. Coupling of the controlling boson and fermionic degree of freedom is asymmetric with ϵ=0.1 in Equation (10). Output intensity Equation (6) for different initial states $|\psi_{i}^{b}\rangle ⊗|N\rangle$ is compared in the (**bottom panel**).

The quantum control implemented by non-classical radiation, represented by a single-mode boson coupled to the fermionic degrees of freedom of a particle in one of the MZ interferometer’s arms, influences the output intensity given by Equation (6), as depicted in Figure 2 and Figure 3 for various initial preparations of the fermionic system: $|\psi_{i}^{b}\rangle$ in Figure 2 and $|\psi_{i}^{a}\rangle$ in Figure 3, respectively, cf. Equation (11). Additionally, in Figure 2, it is assumed that the bosonic control field couples (via spin operator) to both sites of a dimer with the same strength, whereas in Figure 3, the coupling is asymmetric with ϵ=0.1 in Equation (10).

It is crucial to highlight the distinctive features of quantum control. The presence of the controlling system reduces the overall output intensity, akin to the effect observed with classical gate potential. However, the impact is non-monotonic, as evidenced by the middle panels of both Figure 2 and Figure 3. In other words, there exists an optimal value of κ such that the coupling becomes either most or least significant for a given output intensity value. In other words, optimal coupling can be inferred from the middle panel of Figure 1 indicating information leakage which occurs solely in one of two arms of the interferometer. Subsequently, the output intensity Equation (6) decreases.

The output intensity given by Equation (6) is sensitive to the boson’s initial preparation. Here, we focus on number states |N〉 and symmetric coupling. A significant dependence of the output intensity can be observed, as depicted in the bottom panel of Figure 2. Increasing *N* leads to nearly complete damping P→0.5. This effect originates from both information leakage due to fermion–boson entanglement and decreasing overlap in Equation (6) of bosonic components of interfering states.

However, this is not the case for asymmetric coupling, as illustrated in the bottom panel of Figure 3. In such a scenario, the interference pattern allows for discrimination between only two cases: the vacuum N=0 and the excited N>0 states indicating qualitative difference between the two cases. For N=0, the damping of the output intensity Equation (6) is due to bosonic quantum (vacuum) fluctuations only, whereas for N>1, a significant portion of information (resulting in boson–fermion entanglement) becomes transferred from the fermionic dimer to the controlling bosonic field.

## 4. Discussion

Nanosystems comprise particles undergoing various, often highly intricate, interactions and relationships. Some of these interactions are electrostatic or magnetic, while others arise from the indistinguishability of the constituents. The Hubbard-type dimer encompasses all of these aspects. It represents the simplest fermionic system operating within a sector of the Fock space $H_{F}$ which is limited to a direct sum of antisymmetric tensor products of maximally four single-particle Hilbert spaces H:

(15) $$\begin{array}{c} H_{F}=\left\{\mathrm{vacuum}\right\}\overset{4}{\underset{i=1}{⨁}}{\left(⋀H\right)}^{i} \end{array}$$

With Coulomb and magnetic interactions included in its Hamiltonian [2], the Hubbard dimer, despite its simplicity, can serve as a natural building block of realistic many-body systems [3,24]. It offers numerous advantages in using quantum systems with fermionic internal degrees of freedom in interferometric experiments. Not only can one infer fermionic characteristics from the interference pattern but also glean insights into the properties of other systems used to control the interference process.

In this paper, we pursued both of these objectives. We demonstrated how the presence of the Coulomb interaction (both on-site and inter-site) in a fermionic dimer can manifest in the characteristics of the output intensity given by Equation (6). Additionally, we related these properties to the internal structure of the fermionic Hilbert space. Although we worked solely in a two-particle sector of the Fock space, as given by Equation (15), we highlighted the significant role played by the total spin of the initial preparation of the fermionic molecule. This feature becomes even more essential when considering non-classical controls resulting from the coupling of the bosonic field to the dimers’ spin. Our general result indicates that fermionic molecules prepared in a spin-less state $|\psi_{i}^{a}\rangle$ (as per Equation (11)) are significantly less sensitive (less ’controllable’) compared to $|\psi_{i}^{a}\rangle$. Furthermore, the effect of quantum control is non-monotonic with respect to the coupling strength κ in Equation (10). This observation may be crucial for the proper design of experiments confirming our predictions or in other applications of fermionic systems driven by non-classical radiation.

In particular, since interferometry is frequently used in various optoelectronic measuring devices, the predictions reported in this work can serve as guidelines for either inferring the electronic properties of fermionic systems encoded in their Hamiltonians or, through the proper adjustment of non-classical control fields, determining quantum optical features of light, such as its quantum state. Moreover, electronic transport in multiple connected samples, which is strongly influenced by the topology of the samples, is sensitive to the interference of various homotopically inequivalent electron paths. This sensitivity is potentially important in spintronics involving fermionic spin carriers.

The relative richness of the simplest fermionic systems can also be considered a natural drawback, as their controllability stems from a non-trivial interplay of various characteristics that cannot be easily separately designed. This conclusion, supported by the results of our paper, may play a crucial role in potential applications of fermionic systems, both in interferometry and beyond. Understanding the intricate dynamics and dependencies within these systems is essential for effectively harnessing their capabilities in practical applications.

## Data Availability

Data is contained within the article.
